# Percutaneouse ultrasound‐guided needle aspiration for management of breast abscesses – a review

**DOI:** 10.1002/jmrs.682

**Published:** 2023-04-28

**Authors:** Hadeel Ghunaim

**Affiliations:** ^1^ Taibah University Madina Saudi Arabia

**Keywords:** Breast abscess, incision and drainage, lactation, ultrasound‐guided aspiration

## Abstract

Breast abscesses are still a common cause of morbidity among lactational females. Over the years, there has been an increase in the incidence of non‐lactational breast abscesses and a decrease in lactational breast abscesses. The management could be the use of the conventional method of surgical incision and drainage or the newer techniques of needle aspiration or suction drain or catheter in addition to the administration of antibiotics. The use of needle aspiration as the minimal‐invasive conservative technique is generally recommended for abscesses less than 3–5 cm in diameter. However, recent studies have compared the two methods for abscesses larger than 3 cm and among patients with risk factors for breast abscesses. We aim to present the clinical evidence showing the comparison between needle aspiration and incision and drainage for breast abscesses irrespective of the size of the abscesses. There is a lack of comparative information on the two treatment modalities for breast abscesses larger than 3 cm in diameter; however, needle aspiration is being tried because of its advantages like cosmetic preference, short hospital stay and healing time, and no stoppage of breastfeeding.

## Graphical abstract

Breast abscesses are a common cause of morbidity. Management includes the use of the conventional surgical incision with drainage and minimal‐invasive techniques of needle aspiration. Reviewing present clinical evidence to compare between them, needle aspiration considered more advantageous.

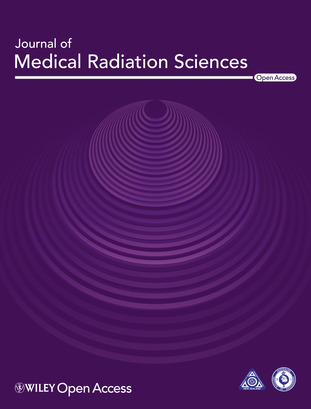

## Introduction

Breast abscesses are one of the common causes of morbidity and interruption of breastfeeding among lactating women.^1^, ^2^, ^3^ Approximately 0.4% of lactating women develop breast abscesses, and 3%–11% of women with mastitis may develop lactational breast abscesses.^4^, ^5^, ^6^, ^7^, ^8^ Although the incidence of breast abscesses is low and is further decreasing in developed countries, these are still common causes of morbidity among women in developing countries.^9^, ^10^, ^11^ The incidence of lactational breast abscesses seems to be decreasing, with a corresponding increase in the incidence of non‐lactational abscesses.^10^, ^12^ Breast abscesses are most commonly caused by infections around the nipple due to biting by the child during feeding; bacterial colonisation due to improper nursing techniques, milk stasis, lactational breast inflammation, lack of proper hygiene and poor economic living, and delay in getting appropriate treatment.^1^, ^2^, ^3^, ^12^ Emergency treatment of breast abscesses might be required to restore normal feeding among lactational females and also to avoid the onset of complications.^1^

The traditional management of breast abscesses included surgical incision and drainage (I&D); however, over time, aspiration with a needle or catheter drainage or suction drainage, or vacuum‐assisted biopsy has replaced I&D, especially for breast abscesses smaller than 3 cm in diameter.^2^, ^3^, ^4^, ^14^, ^15^, ^16^, ^17^ One of the initial comparator clinical studies comparing needle aspiration (NA) with I&D was published more than 25 years ago.^18^ In the early years of this century, the emergency medical treatment guidelines for breast abscesses concluded that NA might be considered an effective first‐line treatment in small breast abscesses.^19^ However, there are lack of guidelines and algorithms for the management of breast abscesses for larger sizes. Additionally, the frontline healthcare workers are not fully trained in the protocols for allocating the patients into different treatment groups.^3^

Both Ultrasound (US)‐guided NA and I&D have been compared in various clinical studies and reviews.^8^, ^20^, ^21^, ^22^ Management with NA is associated with a lower incidence of recurrence, low cost, and better cosmetic results, especially for abscesses <3 cm in diameter.^9^, ^15^, ^20^, ^21^ However, repeated aspirations and the large size of the breast abscess have been considered possible limiting factors. However, I&D is the traditional, invasive, and frequently used method for larger breast abscesses; it requires the use of general anaesthesia, the healing time is prolonged, regular dressings are required, restoration of breastfeeding is difficult, patients are unsatisfied with cosmetic outcomes as there is a high chance of wound granulation, there is a risk of rupture and recurrence of breast abscesses (10%–38%), and lastly, the increased cost is a financial burden on the patient.^1^, ^9^, ^13^

Although the comparative studies and reviews have shown the benefits of NA compared to I&D for small (<3 cm in diameter) abscesses, there is a need to compare the management techniques, especially for larger breast abscesses, and to help formulate a management algorithm according to the presentation of breast abscesses.^8^, ^20^, ^21^ We aimed to evaluate the currently available clinical evidence for the management of breast abscesses of different sizes and identify the limitations and strengths of these clinical studies for their future use in formulating treatment algorithms.

## Characteristic features of breast abscesses

Lactational breast abscesses are generally located in the peripheral breast, and these are commonly reported among women having their first pregnancy, and generally occur in the second or third decade, while the non‐lactational breast abscesses are mainly of sub‐areolar type and generally occur toward the end of the reproductive years.^1^, ^2^, ^7^, ^10^, ^11^, ^14^, ^21^, ^23^, ^24^ The most common causative organism is *Staphylococcus aureus*; however, Methicillin‐Resistant *Staphylococcus aureus* (MRSA) is also increasing the disease burden, followed by *Staphylococci epidermidis* and *Streptococcus*.^1^, ^6^, ^7^, ^8^, ^10^, ^14^, ^23^, ^25^ The most common site of breast abscesses is the upper outer quadrant, and some studies have shown a higher occurrence in the left breasts.^1^, ^7^, ^14^, ^23^ As mothers with cracked or sore nipples usually avoid breastfeeding from the affected side, there is a high risk of milk stasis, which further becomes another cause of breast infection. In addition to the signs of mastitis, like pain, warmth, redness of breasts, asymmetry of breasts, and difficulty in feeding, a fluctuant mass may also be present in patients with breast abscesses.^13^, ^16^, ^24^ Non‐lactational breast abscesses often have a chronic course and have a higher risk of developing into fistulas.^21^ Both lactational and non‐lactational breast abscesses generally need to be drained or aspirated.

## Management of breast abscesses

The general measures include using analgesics, antibiotics, breast support, breast emptying (through pumping or nursing), and continuation of breastfeeding among lactating females.^6^, ^8^, ^12^, ^14^ Patients are generally instructed to continue breastfeeding from the unaffected breast, and the affected breast is routinely emptied with pumps.^14^ US should be performed to identify the location and pockets of the abscess. The specific measures include the drainage or aspiration of the abscesses, which can be either by the NA technique (performed with or without US guidance) or the I&D of the abscess.^6^ A patient is considered clinically recovered when no fever, no breast tenderness, or erythema is present.

### Needle aspiration

Needle aspiration can be performed with or without US guidance. US‐guided aspiration assists in locating the breast abscesses, and in needle positioning and thus enables visualisation of loculi and the adequacy of drainage.^8^, ^9^, ^14^ After local anaesthesia, the needle is inserted (can be in the range of 14–18 G, and a 20 mL syringe), and the pus is drained with syringes.^9^, ^24^ The aspiration procedure is carried out until no further pus can be aspirated, and then the aspiration is repeated daily or on alternate days for the coming days.^8^, ^9^ If the abscess does not resolve after 5 aspirations, then it is considered a case of treatment failure, and another treatment modality is planned.^24^

### Incision and drainage

An incision is made at the site of maximum fluctuation, the whole of the abscess cavity is evacuated, loculi are broken, and, lastly, the wound is packed.^14^ Patients need frequent dressings and proper cleaning.^6^, ^21^

## Clinical studies evaluating various techniques for management of breast abscesses

Since the introduction of NA for breast abscesses, various comparisons have been made in retrospective or prospective clinical studies.^1^, ^2^, ^4^, ^7^, ^9^, ^10^, ^11^, ^12^, ^13^, ^14^, ^23^, ^24^, ^25^, ^26^, ^27^, ^28^, ^29^, ^30^, ^31^, ^32^, ^33^, ^34^, ^35^, ^36^, ^37^ However, the clinical studies not only differ in study designs but also have different baseline characteristics. We did not limit our inclusion criteria based on clinical study design, duration of symptoms, or quality of clinical studies.

We used MEDLINE, Google Scholar, and The Cochrane Library as sources for evidence. Additional studies were searched using the reference lists from the clinical trials and reviews. The details of the clinical studies can be found in Table 1.

**Table 1:** Characteristics of the clinical studies.

Study	Study design and intervention	Participants	Abscess size	Results
Berna‐Serna et al. (2003)^10^	Retrospective study of patients with breast abscesses being treated with percutaneous NA	Patients (*n* = 39) lactating and non‐lactating	NA was carried out when the maximum diameter was ≤3 cm, and catheter drainage was planned for abscesses >3 cm in diameter	There was no significant difference in the resolution of breast abscesses with NA or catheter drainage, and a similar percentage of patients needed repeat aspiration or surgery after NA or catheter drainage, respectively
Colin et al. (2019)^24^	Retrospective analysis to determine recovery with US‐guided procedures without surgical I&D for lactational breast abscesses. Single arm study	Patients (*n* = 95) Total of 105 abscesses and a total of 202 US‐guided percutaneous procedures	The median diameter of abscesses was 4.5 cm, 78% were more than 3 cm, and 38% were larger than 5 cm; the choice of the technique for NA did not have any cut‐off according to the size of the abscess	The US‐guided treatment was successful in 96% of cases, irrespective of the size of the abscesses. 53% of patients needed more than one drainage, and 4% had to undergo surgery under general anaesthesia. 91% of patients continued breastfeeding after NA
Chandika et al. (2012)^9^	A randomised open‐label controlled clinical trial to establish whether US‐guided NA is a feasible alternative treatment for breast abscesses	Patient (*n* = 65) were divided into NA group (*n* = 33) and I&D group (*n* = 32)	Breast abscess size ≤5 cm in diameter	66.2% patients were lactating; there was no difference in breast abscess healing rate by survival analysis (Log rank 0.24 df1; *P* = 0.63). I&D was costlier compared to NA treatment
David et al. (2018)^12^	Retrospective analysis of cases over 12 years to compare NA with I&D as the initial treatment option for breast abscesses	127 abscesses in 114 patients	Mean diameter of the breast abscesses was 3.8 cm	87 abscesses were >3 cm in diameter; 57 abscesses contained gram‐positive organisms. Smoking (*P* = 0.021), and the presence of nipple rings (*P* = 0.005) were significantly correlated with the repeat procedure
Dayal et al. (2019)^11^	A prospective randomised controlled clinical trial to compare US‐guided NA and I&D	Patient (*n* = 100) were divided equally into NA and I&D groups	The size of the abscesses is not mentioned	The average pain score was significantly less in the NA group (VAS score 4.22) compared to the I&D group (VAS score 5.72) on day one. The difference continued till day 14. The duration of hospital stay was 3.08 and 2.48 days in NA and I&D groups respectively
Dener et al. (2003)^7^	A prospective study among lactating patients with breast abscesses and the outcomes were compared between the	Lactating patients (*n* = 26); US‐guided NA (*n* = 10) and I&D group (*n* = 16)	The size of the abscesses is not mentioned.	The mean healing time was shorter in the NA group (*P* = 0.22). *S. aureus* was isolated from 38% cases of breast abscesses. Recurrence rates were 10% and 6.2% in NA and I&D groups respectively
Ding et al. (2020)^25^	A retrospective study of patients treated with US‐guided NA and a comparison of characteristics of all breast abscesses caused by MSSA and MRSA	Lactating patients; MSSA (*n* = 132) and MRSA (*n* = 39)	Abscesses >5 cm in diameter were also included	There were no significant differences in abscess cavity location, abscess cavity amount, and abscess cavity size in the two groups (MRSA and MSSA groups)
Eryilmaz et al. (2004)^13^	Prospective randomised clinical study to compare the effectiveness of NA with I&D	Lactating (*n* = 45) patients; NA (*n* = 22) and I&D (*n* = 23)	The mean abscess size was 6.9 ± 2.7 cm (I&D) group and 6.1 ± 2.8 cm (NA group)	The mean healing time was significantly longer in the I&D group; 70% of patients of the I&D group did not like cosmetic outcome; 41% of the NA group needed I&D
Egbe et al. (2020)^4^	Observational prospective single arm clinical study to estimate the incidence of lactational breast abscesses and describe the management by percutaneous aspiration	*N* = 25	The size of the abscesses is not mentioned	44% of the patients required 3 aspirations, and 24%–28% of patients required 8–9 days for resolution of abscesses 76% of patients continued breastfeeding after NA
Elagili et al. (2007)^28^	Prospective clinical study to assess US‐guided NA as an alternative to surgical I&D	31 abscesses in 30 patients; lactating (14) and non‐lactating (16)	Median diameter of the abscesses was 4 cm (range 1–15 cm)	50% of the patients had complete resolution after one aspiration, multiloculation was significantly related to treatment failure (*P* = 0.002)
Fardhus et al. (2018)^23^	A prospective study to compare the outcome and effectiveness of I&D with NA in the treatment of breast abscesses	50 female patients; equally divided into NA group and I&D group	The mean diameter of the abscesses was 5.7 cm	Mean time taken for the procedure for the NA group (6.62 ± 1.5 min) was significantly shorter than the I&D (18.81 ± 2.10 min) group. A significant difference (*P* = 0.001) in cosmetic outcomes was also reported. Pain and fever get relieved faster at day10 in the NA group (96%) compared to the I&D group (92%). No recurrence in the NA group compared to 3.3% in the I&D group
Giess et al. (2014)^26^	A retrospective review of the database (3 years) of the patients who had undergone aspiration with or without I&D for breast abscesses	A total of 41 abscesses (40 patients)	Mean diameter of the abscesses for the NA group: 4.3 cm (range 0.9–10 cm) Mean diameter of the abscesses of NA + I&D groups: 4.1 cm (range 2.2–7.5 cm)	Out of total, 22 abscesses were treated with NA only, and 19 required aspirations with I&D. Out of the latter group, 15 abscesses were >3 m in diameter; the reasons for I&D included: lack of clinical improvement and recurrence; and fistula development
Hussain et al. (2018)^29^	A comparative analysis of percutaneous aspiration and I&D procedures for the management of breast abscesses	Lactating women; (*n* = 90); divided into two groups, NA (*n* = 45) and I&D (*n* = 45)	Abscesses ≤3 cm were included	Breastfeeding was restored significantly early in the NA group (*P* = 0.011) compared to the I&D group
Li and Ma (2021)^30^	A retrospective clinical study to analyse the risk factors of US‐guided fine‐needle aspiration treatment failure	Patient (*n* = 1472) Lactational breast abscesses	Abscess size is not mentioned	Risk factors for treatment failure included: abscess in the centre area, volume of pus >50 mL, frequency of aspiration >3 times, and treatment time > 14 days
Karvande et al. (2016)^1^	A prospective clinical study to compare outcome and effectiveness of NA and I&D	Patients (*n* = 60) were equally divided into NA and I&D groups	Abscesses <10 cm was included; mean diameter was 5.7 cm	Mean healing time was significantly shorter in the NA group (4.27 days) compared to the I&D group (7.60 days, *P* = 0.001). No recurrence in NA compared to 3.3% in I&D. The time taken for procedure significantly less in the NA group compared to the I&D group (*P* = 0.001). Pain and fever get relieved faster at day10 in the NA group (96.7%) compared to the I&D group (93.3%). Cosmetic outcome was significantly (*P* = 0.001) good in patients treated with NA compared to I&D. Failure seen in 13.3% of patients in the NA group and 3% in the I&D group. No recurrence in the NA group compared to 3.3% in the I&D group
Naeem et al. (2012)^31^	To compare the US‐guided NA with I&D in the treatment of breast abscesses.	Patients (*n* = 64) were equally divided into US‐guided NA group and I&D group	Abscesses <5 cm were included	The mean healing time was significantly less (*P* = 0.001) in the NA group compared to the I&D group. The healing rate was also higher in the NA group; breastfeeding was interrupted in the I&D group
Patil and Vagger (2019)^32^	A prospective non‐randomised controlled trial to compare US‐guided NA and I&D in treating breast abscesses. Lactating female patients	Patient (*n* = 100) were equally divided into the US‐guided NA group and the I&D group	Abscess size <5 cm	The hospital stay was longer in the I&D group; breastfeeding was interrupted in the I&D group; the mean pain score was less in the NA group.
Randhawa et al. (2019)^2^	A randomised controlled clinical trial to compare the frequency of success rate of multiple needle aspirations under antibiotic cover against I&D of breast abscesses	70 female patients were equally divided into NA and I&D groups	The size of the abscesses is not mentioned	The success rate in the NA group was 97.1% compared to 82.9% (*P* = 0.046) in the I&D group
Rigourd et al. (2022)^27^	A retrospective study of management of breast abscesses among lactating females with US‐NA to suggest an algorithm	28 lactating female patients	Size of the abscesses which underwent US‐NA: 4.1 ± 1 cm Size of the abscesses which underwent I&D: 3.9 ± 2 cm	75% were managed by NA and the rest 25% were referred for surgical drainage All patients managed with NA continued breast feeding
Saeed et al. (2021)^33^	To compare the outcome and effectiveness of NA with I&D for breast abscesses	Lactating and non‐lactating women (*n* = 50) were divided equally into two groups on NA and I&D	Abscess size ≤5 cm	The mean healing time was 8.59 + 1.89 and 18.16 + 5.00 days in NA and I&D groups respectively. Good cosmetic outcome in patients treated with NA (no scar) compared to (scar) in the I&D group
Sarhan and Ibraheem (2012)^34^	A prospective study to evaluate the clinical outcome of US‐guided percutaneous NA	Patients (*n* = 43) with breast abscesses	The average size of the abscesses was 2.8 cm (range 0.8–7 cm)	53.4% showed resolution after one aspiration; and 7% required surgical I&D
Sushel et al. (2020)^35^	A cross‐sectional study to evaluate the clinical outcome of US‐guided percutaneous NA	Patients with lactational breast abscesses (*n* = 68)	The size of the abscesses ranged from 2 to 10 cm	73.5% of abscesses responded to US‐guided NA along with antibiotics. 26.4% underwent I&D after failure with NA
Suthar et al. (2012)^36^	A randomised clinical study to compare the management of puerperal breast abscesses by percutaneous US‐guided NA versus open surgical drainage	70 lactating female patients were randomly divided into US guided NA (*n* = 35) and I&D groups (*n* = 35)	The mean abscess size was 4.97 ± 2.5 cm	A failure rate of 17.14% was reported in NA group; resolution time was less in the NA group. Fistula formation was main complication in the I&D group (10%)
Singh et al. (2012)^37^	A prospective single‐arm study to evaluate the treatment of breast abscesses with NA and antibiotics	Lactating (*n* = 31) and non‐lactating (*n* = 19) female patients	78% of patients had breast abscesses of 4 cm, and 22% of cases had a size >4 cm	78% of the abscesses resolved without recurrence. 16% needed I&D after failure with NA
Sikander et al. (2020)^13^	A randomised prospective study to compare the outcome of percutaneous NA with I&D	Lactating patients (*n* = 80); equal number in each group	Patients with breast abscess size <4 cm were included	Cessation of breastfeeding was significantly (*P* < 0.05) less in the NA group; the healing rate was comparable in the two groups; and there was 90% and 100% resolution of abscess in the NA and I&D groups, respectively, after first intervention

I&D, incision and drainage; MRSA, methicillin‐resistant *Staphylococcus aureus*; MSSA, methicillin‐sensitive *Staphylococcus aureus*; NA, needle aspiration; US, ultrasound; VAS, visual analogue scale.

## Clinical evidence

### Outcome measures

The reviewed clinical studies have included success rates, mean healing time and effect of breastfeeding, and cosmetic effects as the major outcome measures. Other outcome measures include the time taken for procedure and hospital stay, post‐intervention symptoms, the incidence of complications, and cost analysis.

#### Success rate

The success rate (absence of symptoms and resolution of pus) has been widely used as an outcome measure in most of the clinical studies. Complete resolution of the abscess was considered a successful treatment with NA.^14^ Most of studies have reported higher success rate with US guided procedure (Table 1). In one of the recent landmark clinical trials by Colin et al.,^24^ US‐guided percutaneous drainage included NA and/or use of pigtail catheter. Colin et al. found that 92% (37/40) of lactational abscesses larger than 5 cm were successfully treated with US‐guided procedure as a first‐line treatment, and none of the cases needed any surgery. The number of percutaneous NA procedures per abscess ranged from 1 to 6 in the study.^24^ Additionally, abscesses of >3 cm and advanced abscesses in a pre‐fistula stage were also successfully treated with one or serial aspirations.^24^ Retrospective analysis by Giess et al.^26^ shows that there was no statistically significant difference in the success of the treatment based on the abscess size (Fisher's exact test, *P* = 0.727). Approx. 72.2% of abscesses among lactating and pregnant women and only 39.1% of the abscesses among non‐lactating and non‐pregnant females responded to NA treatment (Fisher's exact test, *P* = 0.058).^26^ Similar results were reported by Karvande et al., as a success rate of 86.6% was achieved in the NA group. The mean size of breast abscesses (5.58 cm) in the NA group was significantly larger than in the I&D group (*P* = 0.028).^1^ Karvande et al.^1^ also reported that US‐guided NA has great value even in the treatment of breast abscesses with large volumes as approx. 77% of the patients were successfully treated with two aspirations. Another study of US‐guided NA by Kielar et al.^16^ showed that 21 out of 22 cases healed without complications, and the patients could breastfeed and there was no milk retention. Fardhus et al. observed an 88% success rate in the US‐guided NA group and Rigourd et al. reported that 64.3% patients were managed with one inspiration, and the rest needed two (28.6%) or three (3.6%) punctures.^23^, ^27^ Additionally, there was no significant difference in the mean amount of pus drained and the diameter of the abscesses.^22^, ^26^ Suthar et al.^36^ have reported an 82.86% success rate in the US‐guided NA group. Chandika et al.^9^ mention 93.1% as a success rate as only 6.9% of patients were re‐aspirated, and no abscess was converted to the I&D group from the NA group. Sikandar et al.^13^ report that 5% of patients from the NA group needed re‐aspiration, 5% had to undergo I&D, and the rest of the 90% had successful treatment. Eryilmaz et al. reported that a mean of 3.5 (range 1–5) aspirations were performed in the NA group; 45% of patients required multiple aspirations, and I&D technique was used among 41% of patients after failure with NA technique.^14^ Although Eryilmaz et al.^14^ had conducted NA without US guidance, NA was successful among all patients with abscess diameter smaller than 5 cm; and it was also successful among 25% of patients with abscesses larger than 5 cm in diameter (*P* < 0.05). Randhawa et al.^2^ found that both NA and I&D techniques led to successful results (*P* = 0.046) among both lactating and non‐lactating female patients. Ding et al.^25^ reported no significant difference between the methicillin‐sensitive Staphylococcus aureus (MSSA) and methicillin‐resistant Staphylococcus aureus (MRSA) groups regarding abscess sites, number, and size of abscess cavities. Although all patients were treated with US‐guided aspiration, and no comparison could be drawn with I&D, the results show that all the abscesses were treated successfully, irrespective of the size of the abscesses.^25^ Sushel et al.^35^ show that the number of aspirations increases with the increase in the size of the abscesses. Abscess sizes in all non‐responders were >5 cm in diameter.^35^ Berna‐Serna et al.^10^ found that 86.4% of the patients who underwent US‐guided NA had been relieved from acute symptoms within 24–48 hours of the drainage. Elagili et al.^28^ reported a success rate of 83.3% with NA and 13.3% were referred for surgical I&D. Similarly, Singh et al.^37^ also mention that there was no treatment failure with NA for abscesses of 4 cm diameter, whereas 7 out of 11 abscesses with sizes >4 cm I&D had to be performed after failure with NA.

Other studies have shown lower success rates with NA (Table 1), as Saeed et al.^33^ have reported a failure rate of 20% in the NA group and no failure in the I&D group. Egbe et al.^4^ found that 44% of participants from the NA group (without US‐guidance) were aspirated three times, and in 24%–28% of patients, the abscesses resolved in 8–9 days. Singh et al.^37^ found no treatment failure in cases with abscesses of 4 cm, and 64% of those abscesses larger than 4 cm had a treatment failure. Mean number of aspirations required for all patients was 3, (ranging from 1‐to 5).^37^ The success rate was 78% with NA, 16% needed I&D, and the remaining 6% had a recurrence of abscesses.^37^

#### Mean healing time

Healing time for I&D has been defined as the time from incision and drainage to wound closure.^14^ Several clinical studies have reported significant shorter mean healing time with NA compared to I&D (Table 1). Significant differences were found by Karvande et al. (*P* = 0.001), Eryilmaz et al. (Mann–Whitney *U* test, *P* < 0.001), and Saeed et al. (*P* = 0.001).^1^, ^14^, ^33^ However, the comparison will be biased as they did not include 41% of the patients in analysis from the NA group who had treatment failure.^14^ Similarly, Suthar et al.^36^ found the mean healing time to be significantly shorter; however, they had excluded the treatment failures from the analysis. Dener et al.^7^ also showed a shorter mean healing time in the US‐guided NA group (8.6 days, range 5–12 days) compared to the I&D group (10 days, range 6–15 days, *P* = 0.22).

Chandika et al. did not find any significant difference in the mean healing rate, as 65.5% from the US‐guided‐NA group and 58.1% from the I&D group were healed by the 3rd visit.^9^ Clinical study by Egbe et al.^4^ report 6–10 days as the resolution time with NA without US guidance respectively. Fardhus et al.^23^ also showed that mean healing time was significantly shorter in the NA group (4.27 days) compared to the I&D group (7.60 days).

#### Breastfeeding

It is crucial to continue breastfeeding for the mother and infant's health as milk stasis is a risk factor for further development of mastitis and breast abscesses.^13^ Additionally, separating the baby from the mother has a psychological effect on the mother.^37^ Breastfeeding should be resumed after aspiration and can be continued even with a drain in place with proper precautions. Although there are concerns about the risk of infection to the infants, only a few clinical reports have suggested transmission of infections to the infants.^39^ However, Most of the clinical studies report uninterrupted breastfeeding in NA with or without US‐guided procedure,^4^, ^13^, ^24^ in addition to studies that compared the NA group with the I&D group^27^, ^29^, ^31^, ^32^ (Table 1). Sikandar et al. reported that a significantly lower number of patients had cessation of breastfeeding in the NA group than in the I&D group (*P* < 0.05). About 66% of patients continued breastfeeding during treatment with NA in Sikander et al. study.^13^ Colin et al.^24^ have also shown that 91% of women continued breastfeeding when patients underwent NA or NA and pigtail catheter drainage for breast abscesses. Egbe et al.^4^ recorded continuation of breastfeeding among 76% of the patients after NA without US guidance. Even early (within 24 hours) restoration of breastfeeding is also significantly more in the US‐guided NA group compared to the I&D group (*P* = 0.011).^29^

#### Time taken for procedure and hospital stay

The shorter hospital stay decreases the separation time between the mother and the child. The use of general anaesthesia for I&D compared to the use of local anaesthesia for NA helps in decreasing the hospital stay. All the clinical studies measuring the duration of the procedure and hospital stay show a lesser time during NA procedures and, shorter hospital stays in the NA group (Table 1).^1^, ^11^, ^23^, ^32^

#### Cosmetic outcomes

There was a significant difference in the level of satisfaction among patients from the NA group and the I&D group (Table 1).^1^, ^23^, ^33^ A clinical study compared the scars with the Manchester scar scale, and the NA group had alower mean score (*P* = 0.0008) than the I&D group.^38^ Patients of the NA group have reported high satisfaction with the aesthetic effect of the treatment, and after three months there were no visible scars or breast deformations.^1^, ^14^, ^16^, ^32^, ^33^, ^38^

#### Post‐intervention symptoms

The duration of pain and fever was less, varying from significant to non‐significant levels in the NA group (Table 1).^1^, ^11^, ^23^ Patients undergoing NA did not have to be separated from their babies thus preventing any psychological disturbances among the lactating females.^37^ Residual abscess was higher in the NA group compared to the I&D group, whereas incomplete wound healing was more prevalent in the I&D group in an analysis by Patil and Vanger.^32^

#### Post‐procedural complications

Complications in either of the techniques include incomplete clearance, recurrence, fistula formation, and milk suppression.

##### Failure of the procedure

Failure was seen in 13.3% of patients in the NA group and in 3% in the I&D group in the study by Karvande et al.^1^ Eryilmaz et al. reported treatment failure of 41% in the NA without US guidance group, and all these patients had abscesses >5 cm diameter.^14^ Duration of symptoms, the volume of pus aspirated, frequency of aspirations (>3), multiloculations, size of the abscesses (>5 cm), and treatment time (>14 days) are considered risk factors for failure.^1^, ^14^, ^28^, ^35^ In contrast, other factors like a cracked nipple, the presence of *S. aureus* in pus culture, history of abscess, and mastitis during earlier periods of lactation are not considered risk factors for failure of treatment.^14^, ^30^

##### Recurrence

Variable recurrence rates have been observed and reported in the clinical studies (Table 1). Recurrence rate was higher in the NA group (10%) compared to 6.2% in I&D groups without significant difference in the study by Dener et al.^7^ Five percent of patients had re‐aspiration, and another 5% of patients underwent I&D from the NA group in the study by Sikandar et al.^13^ However, no secondary intervention was required in the I&D group.^13^ Both Fardhus et al. and Karvande et al. did not find recurrence in the NA group and 3.3% in the I&D group.^1^, ^23^

##### Fistula formation

Reviews and clinical studies have shown that the risk of fistula formation is lower with US‐guided NA.^6^, ^8^, ^29^ A systematic review of milk fistula among lactating mothers shows that milk fistulae are more frequent following I&D than after needle aspirations.^40^ Naeem et al.^31^ have also reported a higher number of complications (milk fistula) in the I&D group compared to the NA group.

#### Cost analysis

Although it is well documented that I&D is costlier than NA, only a few studies have included the financial aspect and found that NA is cost‐effective compared to the I&D method for breast abscesses.^9^

## Discussion

Review of clinical outcomes with US‐guided NA and I&D procedures shows that the former technique is the first choice for breast abscesses <3 cm in diameter. Additionally, recent studies favour the use of US guided‐NA for abscesses >5 cm in diameter as well.^23^, ^24^, ^25^, ^27^ NA with or without US guidance has shown better success rates and cosmetic outcomes, shorter mean healing times and duration of procedures, among abscesses >5 cm in diameter as well.^23^, ^24^, ^25^, ^27^ Early restoration of breastfeeding or non‐interruption of breastfeeding is significantly better in the NA group compared to I&D groups in all studies.^6^, ^11^, ^21^ US‐guided NA has been successful in both MSSA or MRSA‐infected breast abscesses.^25^ I&D is especially considered among cases of recurrences after NA or if NA is not successful because of thick pus or the large size of the abscess.^7^ Review of the available studies also shows that the patients could continue breastfeeding during NA percutaneous treatment. While the patients undergoing I&D need regular dressings, there is a cessation of breastfeeding, and there is a high risk of fistula formation and post‐operative scarring is also observed.^6^, ^8^, ^11^ Breast abscesses have been commonly located in the upper outer quadrant and in the left breasts.^9^, ^14^, ^23^

Some clinical studies have also reported the risk (or predisposing) factors for the occurrence of breast abscesses. Lactation, cracked nipples, milk stasis, primiparous females, first 12 weeks in the post‐partum period, second‐trimester pregnancy, advanced maternal age, recent breast intervention (biopsy or nipple piercing or lumpectomy), subareolar abscess, smoking, immunocompromised state, diabetes, duration of symptoms before treatment, and increasing age have been suggested as the possible risk factors for occurrence and reoccurrence of breast abscesses.^4^, ^7^, ^12^, ^26^, ^28^ Failure of treatment has been linked with smoking, the presence of nipple rings, multiloculation, and abscess size.^12^, ^28^

Additionally, the review brings forth the limitations and strengths of the two procedures. Recent studies highlight the need to stratify the patients according to baseline features like the size of the abscess and viscosity of the pus before deciding the treatment modality. The benefits and risks of NA and I&D should be weighed before opting for one of the two treatment options. The use of NA for abscesses larger than >5 cm may require a higher number of re‐aspirations; however, the duration of hospital stay, time for the procedure, effect on breastfeeding, cosmetic outcomes, and the cost are all favourable for the NA method compared to the I&D technique (Table 1).^1^, ^9^, ^11^, ^23^, ^24^, ^26^ The use of US guidance for diagnosis and during the procedure can help stratify the patients for selecting the treatment method. Irrigation with saline solution and local use of antibiotics helps in decreasing the viscosity of the pus and high concentrations of antibiotics act directly on the microorganisms.^4^ Aspiration of the breast abscess can be successfully done as the initial mode of management in the treatment, but I&D may remain the final resort for non‐responders.

Another interesting aspect of the review includes the update about the newer techniques and management strategies for breast abscesses. Vacuum‐assisted biopsy (VAB), or Mammotome, has been used in cases of failure with NA or as a substitute for NA.^24^, ^41^ Some authors have reported that a Mammotome may be used along with NA if the abscess is too viscous or it is loculated.^42^ Use of catheters for larger breast abscesses can help to decrease the failure rate and hospital stay.^10^, ^15^, ^43^ Other studies have suggested open drainage followed by primary closure with negative suction drain placement as a safe and better alternative to the standard I&D as there is reduced post‐operative pain, shorter hospital stay, and fewer dressings required.^44^ Also, aspiration of the breast abscess through a wide bore cannula can be done, although this technique might require multiple aspirations. No mode of anaesthesia is required, and it can be done on an outpatient department basis. Apart from the newer techniques, proper stratification of patients according to the size of breast abscess, and viscosity of the pus while choosing the management technique for breast abscesses may also improve the outcomes.^24^

The descriptive review has some limitations and strengths. Although decades have passed since the introduction of US‐guided NA for breast abscesses, comparative analysis of the treatment techniques is marred by the limiting factors of the clinical studies. First and foremost, most of the clinical studies have not distinguished lactational and non‐lactational patients as two separate groups.^11^ Most of the clinical studies are retrospective, or open labelled, or single‐arm clinical studies with small sample size.^10^, ^12^, ^24^, ^25^ Additionally, there are differences in the procedures adopted in the clinical trials, as some have used both NA and pigtail catheter, and some used NA with or without US‐guidance.^12^, ^24^ There were no inclusion criteria concerning abscess size and duration of breast abscesses in most of the clinical studies. Many clinical studies had not included breast abscesses with a larger diameter. The strengths include a wider data search without any restriction of time or language. We tried to include clinical studies without restricting language, patient population, and outcome analysis. Overall, the currently available clinical evidence is insufficient to compare the available treatment options for breast abscesses. Thus, we require data from large sample‐sized real‐world studies or randomised controlled clinical trials.

Future directions: There is a need to formulate protocols for treating breast abscesses (both lactational and non‐lactational), according to the presence of various risk factors and comorbid conditions. The algorithm should include the status of all procedures like suction and drainage, and use of pigtail catheter in addition to the commonly used NA and I&D. This should also include the number of recommended reaspirations before considering the failure of treatment with NA. Additionally, there is a need to establish the benefits of local infiltration of antibiotics compared to oral administration. There is also a need to compare the advantages and disadvantages of US‐guided percutaneous catheter drainage treatment modality compared to US‐guided NA and I&D. The current clinical evidence is also deficient in the preference for the US guidance or palpation method. Maintenance of disease and treatment registries in hospitals can help generate clinical evidence.

## Conclusions

The available clinical studies suggest that the percutaneous US‐guided NA, a minimally invasive technique, is associated with a shorter mean healing time and mean duration of hospital stay, higher success rate, acceptable cosmetic results, minimal effects on breastfeeding, acceptable patient satisfaction, and emotional stability among patients with breast abscesses. The review highlights the need to generate comparative data of NA and I&D as treatment modalities for breast abscesses more than 5 cm in diameter. Multi‐centred randomised clinical trials with a larger sample size can help in generating comparative results.

## Conflict of interest

The authors declare no conflict of interest.

## Data Availability

Data available on request from the authors.

## References

[1] Karvande R , Ahire M , Bhole M , Rathod C . Comparison between aspiration and incision and drainage of breast abscess. Int Surg J 2016; 3: 1773–80.

[2] Randhawa SR , Akram M , Akram H , Sajid M . Comparison of needle aspirations and incision and drainage of breast abscesses. J Univ Med Dent Coll 2019; 10: 40–4.

[3] Patani N , MacAskill F , Eshelby S , et al. Best‐practice care pathway for improving management of mastitis and breast abscess. Br J Surg 2018; 105: 1615–22. 29993125 10.1002/bjs.10919

[4] Egbe TO , Njamen TN , Essome H , Tendongfor N . The estimated incidence of lactational breast abscess and description of its management by percutaneous aspiration at the Douala General Hospital, Cameroon. Int Breastfeed J 2020; 15: 26. 32276628 10.1186/s13006-020-00271-2 PMC7146872

[5] Cusack L , Brennan M . Lactational mastitis and breast abscess: Diagnosis and management in general practice. Aust Fam Phys 2011; 40: 976–9. 22146325

[6] Kataria K , Srivastava A , Dhar A . Management of lactational mastitis and breast abscesses: Review of current knowledge and practice. Indian J Surg 2013; 75: 430. 10.1007/s12262-012-0776-1 PMC3900741 24465097

[7] Dener C , Inan A . Breast abscesses in lactating women. World J Surg 2003; 27: 130–3. 12616423 10.1007/s00268-002-6563-6

[8] Irusen H , Rohwer AC , Steyn DW , Young T . Treatments for breast abscesses in breastfeeding women. Cochrane Database Syst Rev 2015; 2015: CD010490. 26279276 10.1002/14651858.CD010490.pub2 PMC9226721

[9] Chandika AB , Gakwaya AM , Kiguli‐Malwadde E , Chalya PL . Ultrasound guided needle aspiration versus surgical drainage in the management of breast abscesses: A Ugandan experience. BMC Res Notes 2012; 5: 1–7. 22226127 10.1186/1756-0500-5-12 PMC3284878

[10] Berna‐Serna JD , Madrigal M , Berna‐Serna JD . Percutaneous management of breast abscesses. An experience of 39 cases. Ultrasound Med Biol 2004; 30: 1–6. 14962601 10.1016/j.ultrasmedbio.2003.10.003

[11] Dayal P , Lal M . A comparative study of outcomes in management of breast abscess by ultrasound guided needle aspiration against incision and drainage. Int J Med Biomed Stud 2019; 3: 117–21.

[12] David M , Handa P , Castaldi M . Predictors of outcomes in managing breast abscesses‐A large retrospective single‐center analysis. Breast J 2018; 24: 755–63. 29781232 10.1111/tbj.13053

[13] Sikander A , Bano HS , Abdullah M , Najam R , Rasheeqa M , Farooq UM . Comparison of outcome between percutaneous aspiration and incision and drainage in cases of lactational breast abscess. Pak J Surg 2020; 36: 135–40.

[14] Eryilmaz R , Sahin M , Tekelioglu MH , Daldal E . Management of lactational breast abscesses. Breast 2005; 14: 375–9. 16216739 10.1016/j.breast.2004.12.001

[15] Ulitzsch D , Nyman MKG , Carlson RA . Breast abscess in lactating women: US‐guided treatment. Radiology 2004; 232: 904–9. 15284435 10.1148/radiol.2323030582

[16] Kielar M , Raczek‐Pakuła K , Waligóra J , Lewczuk A , Woźniak W , Taranta I . Low invasive treatment of breast abscess in lactating women. Polish Journal of Surgery 2009; 81: 225–9.

[17] Tewari M , Shukla HS . An effective method of drainage of puerperal breast abscess by percutaneous placement of suction drain. Indian J Surg 2006;68:330–3.

[18] O'Hara RJ , Dexter SPL , Fox JN . Conservative management of infective mastitis and breast abscesses after ultrasonographic assessment. Br J Surg 1996; 83: 1413–4. 8944458 10.1002/bjs.1800831028

[19] Mackway-Jones K . Towards evidence based emergency medicine: best BETs from the Manchester Royal Infirmary. Aspiration of breast abscesses. Emerg Med J 2004;21:333–8. PMC1756369 14734386

[20] Bing F , Jie L . Ultrasound guided needle aspiration and cavity washing versus incision and drainage to treat breast abscesses: A meta‐analysis. Int J Clin Exp Med 2017: 8656–65.

[21] Boakes E , Woods A , Johnson N , Kadoglou N . Breast infection: A review of diagnosis and management practices. Eur J Breast Health 2018; 14: 136. 30123878 10.5152/ejbh.2018.3871 PMC6092150

[22] Lam E , Chan T , Wiseman SM . Breast abscess: Evidence based management recommendations. Expert Rev Anti Infect Ther 2014; 12: 753–62. 24791941 10.1586/14787210.2014.913982

[23] Fardhus ‐ MD , Sharfuzzaman A , Dewan MN , Kirttania DC , Hasan ASA , Rab JZ . Comparison between aspiration and incision and drainage of breast abscess. J Surg Sci 2018; 22: 11–5.

[24] Colin C , Delov AG , Peyron‐Faure N , Rabilloud M , Charlot M . Breast abscesses in lactating women: Evidences for ultrasound‐guided percutaneous drainage to avoid surgery. Emerg Radiol 2019; 26: 507–14. 31154537 10.1007/s10140-019-01694-z

[25] Ding ST , He XP , Ma XJ , Zhang Y , Liu XX , Qin J . Lactational breast abscesses caused by methicillin‐resistant or methicillin‐sensitive Staphylococcus aureus infection and therapeutic effect of ultrasound‐guided aspiration. Breastfeed Med 2020; 15: 471–4. 32412775 10.1089/bfm.2020.0003

[26] Giess CS , Golshan M , Flaherty K , Birdwell RL . Clinical experience with aspiration of breast abscesses based on size and etiology at an academic medical center. J Clin Ultrasound 2014; 42: 513–21. 24975466 10.1002/jcu.22191

[27] Rigourd V , Benoit L , Paugam C , et al. Management of lactating breast abscesses by ultrasound‐guided needle aspiration and continuation of breastfeeding: A pilot study. J Gynecol Obstet Hum Reprod 2022; 51: 102214. 34469779 10.1016/j.jogoh.2021.102214

[28] Elagili F , Abdullah N , Fong L , Pei T . Aspiration of breast abscess under ultrasound guidance: Outcome obtained and factors affecting success. Asian J Surg 2007; 30: 40–4. 17337370 10.1016/S1015-9584(09)60126-3

[29] Hussain N , Khan I , Ahmed T , et al. Comparison of the restoration of breast feeding after percutaneous aspiration vs incision and drainage for management of breast abscess. J Liaquat Univ Med Heal Sci 2018; 17: 47–51.

[30] Li Y , Ma XJ . Risk factors for failure of ultrasound‐guided fine‐needle aspiration therapy for lactational breast abscess. Breastfeed Med 2021; 16: 894–8. 34165329 10.1089/bfm.2021.0060

[31] Naeem M , Rahimnajjad MK , Rahimnajjad NA , Ahmed QJ , Fazel PA , Owais M . Comparison of incision and drainage against needle aspiration for the treatment of breast abscess. Randomized Controlled Trial 2012; 78: 1224–7. 23089439

[32] Patil SB , Vagger VM . To compare the effectiveness of conventional incision and drainage versus USG guided percutaneous needle aspiration in treatment of breast abscess. J Evolution Med Dent Sci 2019; 8: 1358–61.

[33] Saeed S , Naumani AR , Khalid R , Naqi SA , Kazmi A . Comparison between needle aspiration versus incision and drainage in management of breast abscess. Pakistan J Surg 2021; 37: 56–8.

[34] Sarhan HH , Ibraheem OM . Percutaneous needle aspiration of breast abscesses. J Fac Med Bagdad 2012; 54: 118–21.

[35] Sushel C , Kalhoro N , Luxmi S , Das K , Khaskheli GA , Mallah Q . Outcome of percutaneous needle aspiration in the treatment of lactational breast abscess. J Liaquat Univ Med Health Sci 2020; 19.

[36] Suthar K , Mewada B , Surati K , Shah J . Comparison of percutaneous ultrasound guided needed aspiration and open surgical drainage in management of puerperal breast abscess. Int J Med Sci Public Health 2013; 2: 69.

[37] Singh G , Singh G , Singh LR , Rahul S , Singh S , Lekhachandra KS . Management of breast abscess by repeated aspiration and antibiotics. J Med Soc 2012; 26: 2012–4.

[38] SRH R . Analytical study of drainage of breast abscess by open drainage with primary suturing with negative suction drain and conventional incision and drainage. Int J Surg Sci 2019; 3: 397–9.

[39] WHO 2000 . World Health Organization: Mastitis: Causes and Management. Number WHO/FCH/CAH/00.13. 2000. Available at: https://apps.who.int/iris/bitstream/handle/10665/66230/WHO_FCH_CAH_00.13_eng.pdf?sequence=1&isAllowed=y.

[40] Alipour S , Dinas K . A systematic review of milk fistula in nursing mothers: modifying the perspective toward maintenance of breastfeeding. Clin Lactat 2020; 11: 150–63.

[41] Chen C , Luo LB , Gao D , et al. Surgical drainage of lactational breast abscess with ultrasound‐guided Encor vacuum‐assisted breast biopsy system. Breast J 2019; 25: 889–97. 31148346 10.1111/tbj.13350 PMC6851758

[42] Varey AHR , Shere MH , Cawthorn SJ . Treatment of loculated lactational breast abscess with a vacuum biopsy system. Br J Surg 2005; 92: 1225–6. 15997452 10.1002/bjs.5075

[43] Afridi SP , Alam SN , Ainuddin S . Aspiration of breast abscess through wide bore 14‐gauge intravenous cannula. J Coll Phys Surg Pak 2014; 24: 719–21. 10.2014/JCPSP.719721 25327913

[44] Raj M , Arya S , Chejara RK , Chaudhary R , Mani V . Management of breast abscess by open drainage with primary closure versus standard incision and drainage – a randomised trial. J Clin Diagn Res 2021; 3: 397–9.

